# β-Catenin Directs Nuclear Factor-κB p65 Output *via* CREB-Binding Protein/p300 in Human Airway Smooth Muscle

**DOI:** 10.3389/fimmu.2017.01086

**Published:** 2017-09-08

**Authors:** Tim Koopmans, Roos Eilers, Mark Menzen, Andrew Halayko, Reinoud Gosens

**Affiliations:** ^1^Department of Molecular Pharmacology, University of Groningen, Groningen, Netherlands; ^2^Groningen Research Institute for Asthma and COPD (GRIAC), University of Groningen, Groningen, Netherlands; ^3^Department of Physiology and Pathophysiology, University of Manitoba, Winnipeg, MB, Canada

**Keywords:** β-catenin, CREB-binding protein, p300, airway smooth muscle, nuclear factor-κB, inflammation, chronic airway disorders

## Abstract

β-Catenin is a multifunctional protein that apart from its role in proliferative and differentiation events, also acts upon inflammatory processes, mainly *via* interaction with nuclear factor-κB (NF-κB). However, there is still controversy as to whether β-catenin facilitates or represses NF-κB output. Insights into the molecular mechanisms underlying the interaction between β-catenin and NF-κB have highlighted the cofactors CREB-binding protein (CBP) and p300 as important candidates. Here, we hypothesized that the interaction of β-catenin with CBP/p300 directs NF-κB output. Using human airway smooth muscle (ASM) cells, we found that β-catenin is essential in interleukin -1β (IL-1β)-mediated expression of interleukin-6 (IL-6) by promoting nuclear translocation of the p65 subunit of NF-κB. These effects were independent from WNT pathway activation or other factors that promote β-catenin signaling. In the nucleus, inhibition of either the CBP- or p300-β-catenin interaction could regulate NF-κB output, by enhancing (CBP inhibition) or inhibiting (p300 inhibition) IL-1β-induced expression of IL-6, respectively. Acetylation of p65 by p300 likely underlies these events, as inhibition of the p300-β-catenin interaction diminished levels of acetylated p65 at lysine 310, thereby reducing p65 transcriptional activity. In conclusion, β-catenin is a critical component of NF-κB-mediated inflammation in human ASM, affecting transcriptional output by interacting with the nuclear cofactors CBP and p300. Targeting β-catenin may be an alternative strategy to treat airway inflammation in patients with airway disease, such as asthma.

## Introduction

β-Catenin is a multifunctional protein that plays a fundamental role in the formation and maintenance of tissues, where different cellular compartments serve different needs. At the cell surface, β-catenin is a key component of adherens junctions where it binds the cytoplasmic domain of cadherin transmembrane proteins, allowing cells to engage contact with neighboring cells (1). In the cytosol, a cadherin-free pool of β-catenin functions as a binding partner for a great variety of proteins (2). Cytosolic β-catenin can shuttle between the cytosol and nucleus independent of transporter receptors (3), where it can activate nuclear signaling through the T-cell factor/lymphoid enhancer-binding factor 1 (TCF/LEF1) family of transcription factors to facilitate promoter binding and activation of genes involved in cellular differentiation, proliferation, and migration (4). In the absence of extracellular signals, levels of cytosolic β-catenin are being kept low by means of an axin-adenomatous polyposis coli scaffolding complex that captures β-catenin and subjects it to sequential phosphorylation at serine 45 by casein kinase-Iα followed by phosphorylation at positions 41, 37, and 33 by glycogen synthase kinase (GSK)-3 at the N-terminus, leading to its proteosomal degradation (5, 6). In the presence of extracellular WNT proteins, the destruction complex is sequestered toward the membrane, effectively rescuing β-catenin from GSK-3-mediated phosphorylation, and allowing for its accumulation (7, 8). Alternatively, WNT-independent mechanisms also mediate GSK-3 inhibition and stabilization of β-catenin *via* growth factors such as platelet-derived growth factor (PDGF) and transforming growth factor beta (TGF-β). Protein kinase B/Akt (9, 10), phospholipase C (11), and protein kinase A (PKA) (12) have all been demonstrated to inhibit GSK-3 through phosphorylation of serine 21 in GSK-3α and serine 9 in GSK-3β. Also β-catenin is subjected to phosphorylation by Akt (13, 14) and PKA (15, 16) at serine 552 and 675, increasing its stability and transcriptional activity. In the nucleus, β-catenin pairs up with several partners, among which the transcriptional coactivators CREB-binding protein (CBP) or its closely related homolog E1A-associated protein p300 to assemble a functional transcriptional complex (17, 18).

β-Catenin is well known for its role in cellular homeostasis, regulating cell division, and differentiation. However, β-catenin has also been observed to act upon inflammatory processes, mainly *via* interaction with nuclear factor-κB (NF-κB). These studies mostly point toward an inhibitory role for β-catenin in NF-κB signaling. β-Catenin can physically interact with p65 (19–21) or p50 (19, 20, 22–24) in various cell types, resulting in reduced NF-κB DNA binding, transactivation activity, and target gene expression induced by proinflammatory cytokines such as interleukin-1β (IL-1β) or tumor necrosis factor-alpha (TNF-α). Also GSK-3 has been demonstrated to be required for NF-κB activation, presumably *via* degradation of β-catenin (25–27), although direct phosphorylation of NF-κB p65 by GSK-3 has also been proposed (28, 29). Not all published findings report inhibition mediated by β-catenin. In alveolar epithelial cells, while an increase in β-catenin signaling together with toll-like receptor (TLR) ligand stimulation resulted in reduced NF-κB activation, increased β-catenin signaling in the absence of TLR activation enhanced NF-κB signaling and release of proinflammatory cytokines (30). Similarly, in primary intestinal epithelial cells, stabilization of a degradation-resistant mutant form of β-catenin was associated with elevated NF-κB activation *in vivo* (31). It remains unclear how this disparity is regulated at the molecular level, but recent insights have proposed that the coactivator CBP is involved (31, 32).

Nuclear factor-κB has an important role in airway diseases like asthma and chronic obstructive pulmonary disease (COPD) that are characterized by chronic inflammation. NF-kB regulates the expression of a vast array of cytokines, chemokines, and cell adhesion molecules in the lungs and its activation in asthma and COPD is largely driven by inflammatory mediators like interleukin-1 (IL-1), tumor necrosis factor or by the activation of TLRs during bacterial or viral exacerbation. In addition, a proportion of patients display resistance to glucocorticoids, the mainstay therapy for these conditions, characterized by failure to inhibit NF-kB signaling. This highlights NF-kB as an important candidate target in the treatment of chronic airway disease. However, current NF-κB pathway inhibitors lack specificity, and mechanisms of action are generally not well established. We have recently shown that the airway smooth muscle (ASM) is an important target for β-catenin inhibition (33) *in vivo* and that inhibition of the β-catenin/CBP interaction through the small-molecule compound ICG-001 effectively blocks ASM-driven remodeling in an ovalbumin model for allergic asthma. The ovalbumin mouse model is characterized by strong activation of NF-κB, in part mediated by the immunomodulatory functions of the ASM (34). We also found evidence that disruption of the CBP/β-catenin interaction altered the inflammatory profile in this model. ASM utilize both CBP and p300 (33), and although structurally very similar, they are often assigned opposing roles (35). We hypothesized that in human ASM, CBP and p300 are critical components of β-catenin-mediated NF-κB signaling and we investigated the molecular mechanisms involved in cultured human ASM cells.

## Materials and Methods

### Cell Culture

The cultured human ASM cells used to generate each cell line were obtained from three patients with a diagnosis of adenocarcinoma. The actual resected lung biopsy was considered healthy and pathologically uninvolved according to the clinician’s judgment. Anatomically, segments (0.5–1.0 cm diameter) were obtained from the second to fourth generation main bronchus (36). Informed consent was given by patients for the secondary use of resected material for research purposes. Consent forms and procedures were approved by the Human Research Ethics Board (University of Manitoba, Winnipeg, MB, Canada) in accordance with local and national guidelines. The different donor cells were immortalized by stable expression of human telomerase reverse transcriptase (hTERT). Cells were passaged a maximum number of 30 times for all experiments and were cultured in uncoated 100/20 mm tissue culture dishes (GBO, #664160) in Dulbecco’s Modified Eagle’s Medium (DMEM) (GIBCO, #42430-082) supplemented with 200 units/mL penicillin–streptomycin (GIBCO, #15070-063), 2.5 µg/mL antimycotic (GIBCO, #15290-026), and 10% v/v fetal bovine serum (FBS) (Thermo Scientific, #SV30180.03).

### RT-qPCR

Following a PBS wash, cells were incubated with lysis buffer and subsequently scraped from the plate. mRNA isolation was performed with a NucleoSpin^®^ RNA isolation kit (Macherey-Nagel, #740955.250) in line with the manufacturer’s instructions. cDNA levels were normalized and synthesized using AMV reverse transcriptase (Promega, #A3500), and thereafter diluted 15 times with RNAse-free ddH_2_O. Quantitative real-time PCR was conducted with SYBR green as the DNA binding dye (Roche, #04913914001) on an Illumina Eco Real-Time PCR system. PCR cycling was performed as follows: 94°C for 30 sec, annealing at 60°C for 30 sec, and extension at 72°C for 30 sec, for 45 cycles. RT-qPCR data were analyzed with LinRegPCR analysis software (37, 38). 18S ribosomal RNA, beta-2-microglobulin (B2M), and ribosomal protein L13A (RPL13A) were used as reference loci for accurate normalization of the RT-qPCR data. Primer sequences are listed in Table 1. Total RNA concentrations were determined with a NanoDrop ND-1000 spectrophotometer, and samples were normalized accordingly.

**Table 1:** Primer sequences used.

Amplicon	Species	Forward sequence (5′→3′)	Reverse sequence (5′→3′)
IL-6	*Homo sapiens*	GCCGGGATGGCTTCTATGAG	AGGGGTTGTTGTTGGTCTGG
AXIN2	*Homo sapiens*	CCTGCCACCAAGACCTACAT	CTTCATTCAAGGTGGGGAGA
WNT1	*Homo sapiens*	CCGATGGTGGGGTATTGTGAA	TCCCCGGATTTTGGCGTATC
CTNNB1	*Homo sapiens*	AGGTCTGAGGAGCAGCTTCA	CCATCAAATCAGCTTGAGTAGCC
WNT3	*Homo sapiens*	ACTTCGGCGTGTTAGTGTCC	CCAGGATAGTCGTGCGGC
IL-8	*Homo sapiens*	CAGAGACAGCAGAGCACACA	GGCAAAACTGCACCTTCACA
IL-1β	*Homo sapiens*	TGCTCTGGGATTCTCTTCAGC	TGGAAGGAGCACTTCATCTGTT
18S ribosomal RNA	*Homo sapiens*	CGCCGCTAGAGGTGAAATTC	TTGGCAAATGCTTTCGCTC
B2M	*Homo sapiens*	AAGCAGCATCATGGAGGTTTG	AAGCAAGCAAGCAGAATTTGGA
RPL13A	*Homo sapiens*	ACCGCCCTACGACAAGAAAA	GCTGTCACTGCCTGGTACTT

### Enzyme-Linked Immunosorbent Assay (ELISA)

Twenty-four hours after stimulation, culture medium was collected for the determination of released cytokines, specifically IL-6, using ELISA. Secreted IL-6 was measured with the PeliKine compact™ human IL-6 ELISA kit (#M1916, Sanquin) according to the manufacturer’s instructions. In brief, microtiter plates were coated with anti-IL-6 antibody. The next day, following washing, plates were blocked with 5% BSA w/v in 1× PBS for 1 h at RT. After washing, samples were incubated on the plate for 1 h at RT, after which samples were aspirated and plates were washed again. Plates were incubated with biotinylated anti-IL-6 for 1 h at RT, after which plates were covered with streptavidin-HRP conjugate for 30 min at RT. Next, plates were incubated with substrate solution for 30 min at RT. Finally, the reaction was stopped with stop solution, and absorbance was read at 450 nm.

### WNT-3A Conditioned Medium

Mouse L-cells stably expressing WNT-3A, obtained from ATCC, were used to obtain WNT-3A conditioned medium. Conditioned medium obtained from parental L-cells was used as control. Conditioned medium was prepared as per ATCC guidelines. In brief, cells were grown in DMEM (GIBCO, #42430-082) supplemented with 200 units/mL penicillin–streptomycin (GIBCO, #15070-063), 2.5 µg/mL antimycotic (GIBCO, #15290-026), 0.5 mmol sodium pyruvate (GIBCO, #11360070), 10% v/v FBS (Thermo Scientific, #SV30180.03), and 0.4 mg/mL G418 until confluence after which they were split 1:10 and reseeded in uncoated 145/20 mm tissue culture dishes (GBO, #639160) without G418 for 4 days. Medium was collected, centrifuged at 5,000 rpm for 10 min, and passed through a sterile 0.2 µM filter. New medium was added and cells were grown up to day 7, after which medium was collected as before and subsequently mixed with the previous batch.

### Small-Interfering RNA (siRNA) and DNA Transfection

Airway smooth muscle cells grown to 90% confluence were transfected with 2.5 µg of mutant pcDNA3-S33Y β-catenin plasmid (#19286, Addgene) (39) in plain DMEM using Lipofectamine^®^ LTX with Plus™ Reagent (Invitrogen, #15338) for 24–36 h. 2 µg of green fluorescent protein (GFP) expression vector was transfected as a negative control. Afterward, medium was replaced with plain DMEM and cells were stimulated. For the siRNA constructs, cells were transfected with 30 pmol of p65 siRNA (Invitrogen, #RELAHSS109161) or control siRNA construct (Invitrogen, #BLOCK-iT Stealth RNAi Control) in plain DMEM using Lipofectamine^®^ RNAiMAX™ Transfection Reagent (#13778, Invitrogen) for 24–36 h. siRNA constructs were ordered from Invitrogen. Stealth RNAi™ siRNA Negative Control was used as control siRNA. After transfection, medium was replaced with plain DMEM and cells were stimulated.

### Western Blot Analysis

Following a PBS wash, PBS was aspirated and cells were incubated with RIPA lysis buffer (65 mM Tris, 155 mM NaCl, 1% Igepal CA-630, 0.25% sodium deoxycholate, 1 mM EDTA, pH 7.4, and a mixture of protease inhibitors: 1 mM Na_3_VO_4_, 1 mM NaF, 10 µg/mL leupetin, 10 µg/mL pepstatin A, 10 µg/mL aprotinin), and scraped from the plate. Lysates were kept on ice for 15 min, and thereafter vortexed vigorously and subsequently centrifuged for 10 min at 10,000 *g*. A BCA protein assay kit (Thermo Scientific, #23225) was used to determine protein content of the supernatant fractions. Samples were subsequently subjected to SDS-PAGE, using 8 and 10% running gels (depending on protein size). Separated proteins fractions were transferred on a PVDF membrane (Carl Roth, 0.45 µm, #T830.1), and were then blocked with ROTI^®^-Block blocking solution (Carl Roth, #A151.2) for 2 h at room temperature. Following blocking, membranes were incubated with primary antibodies overnight at 4°C in TBST [50 mM Tris-HCl, 150 mM NaCl, 0.05% (w/v) Tween-20, pH 7.4]. The next day, membranes were washed thoroughly in TBST and then incubated with HRP-conjugated secondary antibody for 2 h at room temperature. TBST was used to dilute the antibodies. Finally, blots were developed using enhanced chemiluminescence substrate (Perkin Elmer, #NEL105001EA). Images were quantified digitally by densitometry using LI-COR Image Studio Lite software.

### Nuclear Fractionation

Following stimulation, cells were washed once with ice-cold 1× PBS and lysed in 300 µL hypotonic Tris-lysis buffer (20 mM Tris, 10 mM NaCl, 3 mM MgCl_2_, pH 7.4, and a mixture of protease inhibitors: 10 µg/mL leupetin, 10 µg/mL pepstatin A, 10 µg/mL aprotinin), scraped and collected in an Eppendorf cup on ice. After 10 min, NP-40 was added to a final concentration of 0.3% and allowed to incubate for 10 min at 4°C under constant rotation. Lysate was then centrifuged at 3,000 *g* for 10 min at 4°C and supernatant (cytosolic fraction) was collected. The pellet was washed once with hypotonic Tris-lysis buffer, after which nuclear proteins were extracted with 50 µL of cell extraction buffer (25 mM HEPES, 420 mM NaCl, 10% sucrose w/v, 10 mM KCl, 1 mM EDTA, 10% glycerol, 10 mM NaF, 10 mM Na_3_VO_4_, pH 7.4, and a mixture of protease inhibitors: 10 µg/mL leupetin, 10 µg/mL pepstatin A, 10 µg/mL aprotinin). After 30 min of incubation and occasional vortexing, lysate was cleared by centrifugation at 14,000 *g* for 20 min at 4°C.

### TOPFlash Assay

Cells grown in DMEM supplemented with antibiotics, antimycotics and 10% v/v FBS were seeded on uncoated six-well plates (Sigma Aldrich, #CLS3506) and grown to 90% confluence. Cells were then transfected in plain DMEM for 24 h with either 1 µg M50 Super 8x TOPFlash (Addgene, #12456) (40) or M51 Super 8x FOPFlash (Addgene, #12457) (40) reporter plasmids using Lipofectamine^®^ LTX with Plus™ Reagent (Invitrogen, #15338). Following transfection, cells were washed with PBS and subjected to stimulation. Luciferase activity was assayed with the Dual-Luciferase Reporter assay system (Promega, #E1910) according to the manufacturer’s instructions.

### Coimmunoprecipitation

Following stimulation, ASM cells were washed once with 1× PBS and then fixed in dithiobis(succinimidyl propionate) crosslinker (Thermo Scientific, #22585) dissolved in 1× HBSS (GIBCO, #14065) up to a final concentration of 1 mM for 30 min at RT. After 30 min, reaction was quenched by incubation with 50 mM glycine dissolved in 1× PBS for 15 min at RT. Next, cells were lysed in RIPA lysis buffer (50 mM Tris, 150 mM NaCl, 1% Triton-X-100, 0.5% sodium deoxycholate, 0.1% SDS, 1 mM EDTA, 10 mM NaF, 10 mM Na_3_VO_4_, pH 7.4, and a mixture of protease inhibitors: 10 µg/mL leupetin, 10 µg/mL pepstatin A, 10 µg/mL aprotinin), scraped and collected in an Eppendorf cup on ice. Lysate was precleared with 50 µL protein A/G bead slurry (#sc-2003, Santa Cruz) for 1 h at 4°C under constant rotation. Beads were spun down at 300 *g* for 1 min and supernatant was cleared by centrifugation at 14,000 *g* for 20 min at 4°C. Protein concentration was measured using a BCA protein assay kit (Thermo Scientific, #23225) and 1,000 µg of protein lysate was incubated with 2 µg antibody overnight at 4°C under constant rotation. The next day, immunocomplexes were incubated with 50 µL of protein A/G bead slurry for 4 h at 4°C under constant rotation. Beads were spun down at 300 *g* for 1 min and washed three times with lysis buffer. Finally, 25 μL of 4× SDS-loading buffer and 25 µL lysis buffer were added to the beads and heated for 5 min at 95°C under constant agitation to reverse cross-linking and elute protein-bead complexes. Following brief spin down, supernatant was subjected to SDS-PAGE.

### Antibodies and Chemicals

The following antibodies were used: NF-κB p65 (western blot 1:1,000, rabbit, Cell Signaling, #8284), GAPDH (Western blot 1:3,000, mouse, Santa Cruz, #sc-47724), phospho (Ser 473) Akt (Western blot 1:1,000, rabbit, Cell Signaling, #9271), Akt (Western blot 1:1,000, rabbit, Cell Signaling, #9272), phospho (Ser21/9) GSK-3α/β (Western blot 1:1,000, rabbit, Cell Signaling, #9331), GSK-3α/β (Western blot 1:1,000, mouse, Santa Cruz, #sc-7291), phospho (Ser675) β-catenin (Western blot 1:1,000, rabbit, Cell Signaling, #4176), β-catenin (Western blot 1:1,000, mouse, BD Biosciences, #610154), Histone H3 (Western blot 1:1,000, rabbit, #4499), non-phospho (active) β-catenin (Western blot 1:500, rabbit, Cell Signaling, #8814), IκB-α (Western blot 1:1,000, rabbit, Santa Cruz, #sc-203), acetyl NF-κB p65 (Lys310) (Western blot 1:500, rabbit, Cell Signaling, #3045), and normal mouse IgG (Santa Cruz, #sc-2025).

Other reagents include the following: DMSO (Sigma Aldrich, #472301), ICG-001 (Tocris, #4505), XAV-939 (Tocris, #3748), IQ-1 (Tocris, #4713), FBS (Thermo Scientific, #SV30180.03), and recombinant IL-1β (Sigma Aldrich, #SRP3083). All other chemicals were of analytical grade.

### Statistical Analysis

All data represent the mean ± SEM, of at least four independent experiments. Owing to unreliable *p* values obtained with small sample sizes, data with an *n* below 4 were not statistically analyzed. A Shapiro–Wilk’s test (*p* > 0.05) as well as visual inspection of the respective histograms, normal Q-Q plots and box plots was used to test whether samples were normally distributed (approximately), using IBM SPSS Statistics version 23. Two group comparisons were made using an unpaired Student’s *t*-test for normally distributed data or a Mann–Whitney *U* test as the non-parametric equivalent. Comparisons between three or more groups were performed using a one-way ANOVA followed by Tukey’s *post hoc* test for normally distributed data, or with a Kruskal–Wallis *H* test for non-normally distributed data. A value of *p* < 0.05 was considered statistically significant. Analyses were performed with GraphPad Prism (GraphPad Software, Inc.).

## Results

### β-Catenin Is Required for IL-1β-Induced Expression of IL-6 in Human ASM

**Figure 1:**
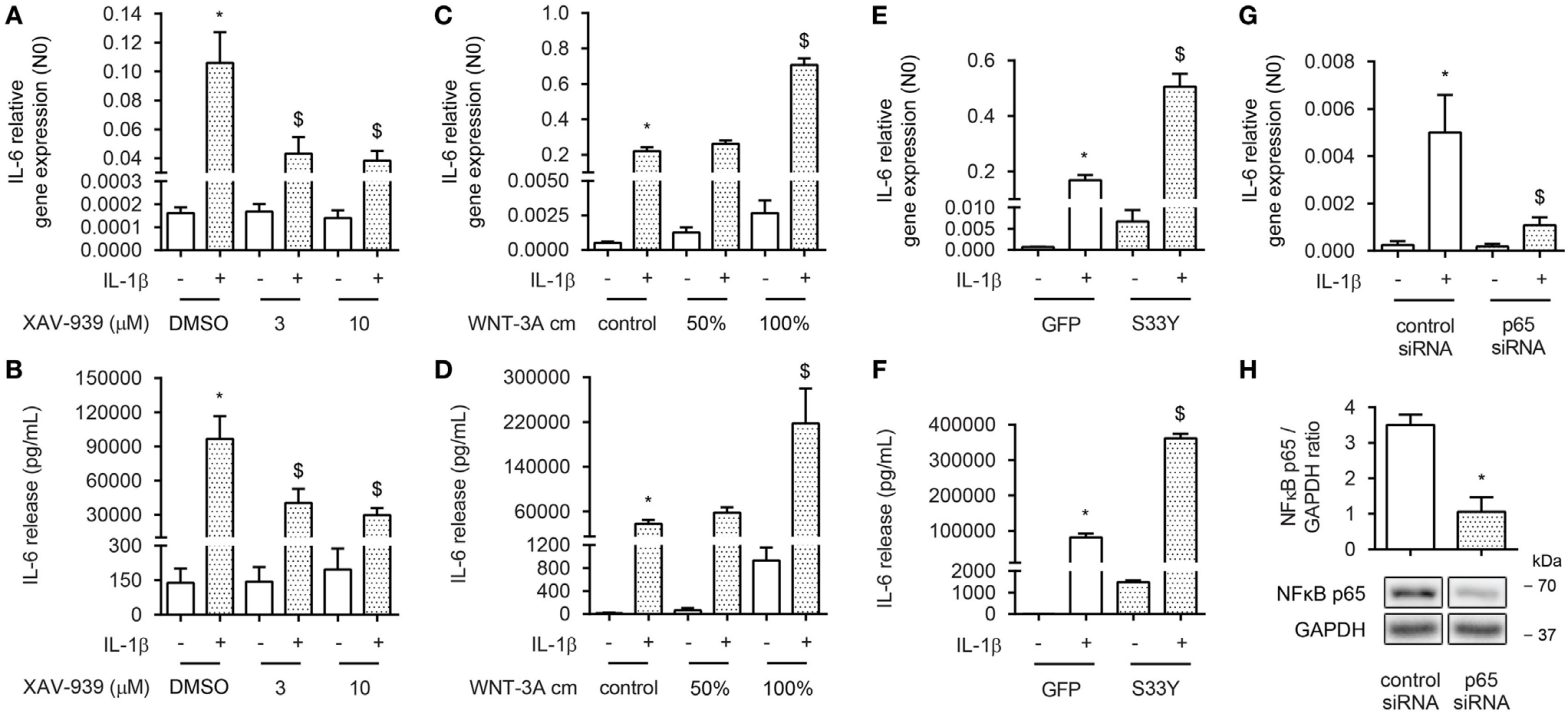
β-Catenin is required for interleukin-1β (IL-1β)-induced expression of IL-6. **(A)** IL-6 mRNA and **(B)** secreted protein from airway smooth muscle cells exposed to IL-1β (1 ng/mL) for 16 h **(A)** or 24 h **(B)** with or without XAV-939 (3 and 10 µM). RNA was isolated and subjected to RT-qPCR **(A)**, whereas cell supernatants were subjected to enzyme-linked immunosorbent assay (ELISA) **(B)**. Data represent four and six independent experiments for **(A,B)**, respectively. **(C)** IL-6 mRNA and **(D)** secreted protein from airway smooth muscle exposed to IL-1β (1 ng/mL) for 16 h **(C)** or 24 h **(D)** with or without WNT-3A conditioned medium. Data represent four independent experiments. **(E)** IL-6 mRNA and **(F)** secreted protein from airway smooth muscle cells exposed to IL-1β (1 ng/mL) for 16 h **(E)** or 24 h **(F)** while transiently transfected with a green fluorescent protein (GFP) or S33Y-construct. Data represent four independent experiments. **(G)** IL-6 mRNA of airway smooth muscle exposed to IL-1β (1 ng/mL) for 16 h while transiently transfected with control or p65 small-interfering RNA (siRNA). Data represent four independent experiments. **(H)** NF-κB p65 immunoblot, normalized against GAPDH. Cells were transfected with 30 pmol control or p65 siRNA for 24 h. Data represent four independent experiments. * vs. control, $ vs. IL-1β.

To assess whether β-catenin is involved in NF-κB-mediated inflammation in ASM cells, we exposed bronchial ASM cells, immortalized by stable expression of hTERT, to the proinflammatory cytokine IL-1β in the absence or presence of the small molecule compound XAV-939. XAV-939 is a WNT-specific β-catenin antagonist that promotes formation of the destruction complex by inhibiting Tankyrase 1 and 2 to stabilize Axin. We have previously shown that XAV-939 treatment results in diminished levels of non-phosphorylated (active) β-catenin in ASM cells (33). We observed that reduced levels of active β-catenin induced by XAV-939 dose dependently inhibited IL-1β-driven expression of IL-6 on both mRNA and protein level (Figures 1A,B), suggesting β-catenin is required for IL-1β signaling. Accordingly, elevated cytosolic levels of β-catenin after treatment with WNT-3A conditioned medium (cm) dose dependently increased IL-1β-driven expression of IL-6 on both mRNA and protein level (Figures 1C,D). Interestingly, WNT-3A cm by itself also enhanced levels of IL-6, albeit to a much lesser extent compared to when IL-1β was included. Similarly, when ASM cells were transiently transfected with an expression vector encoding the degradation resistant S33Y-mutated β-catenin protein, we found dramatically increased IL-6 levels compared to GFP-transfected cells at both mRNA and protein levels (Figures 1E,F). The S33Y-mutant resulted in elevated levels of IL-6 even in the absence of IL-1β. To verify that the observed IL-1β-induced expression of IL-6 was also mediated through activation of NF-κB p65, we inhibited expression of p65 by means of siRNA targeted against p65. This reached a knockdown efficiency of 70 ± 11% (Figure 1H). As expected, p65 siRNA almost completely blocked IL-1β-induced accumulation of IL-6 mRNA (Figure 1G). Taken together, in human ASM, active β-catenin, and NF-κB signaling are essential in the regulation of IL-6 mediated by IL-1β.

### IL-1β Does Not Promote WNT/β-Catenin Signaling

**Figure 2:**
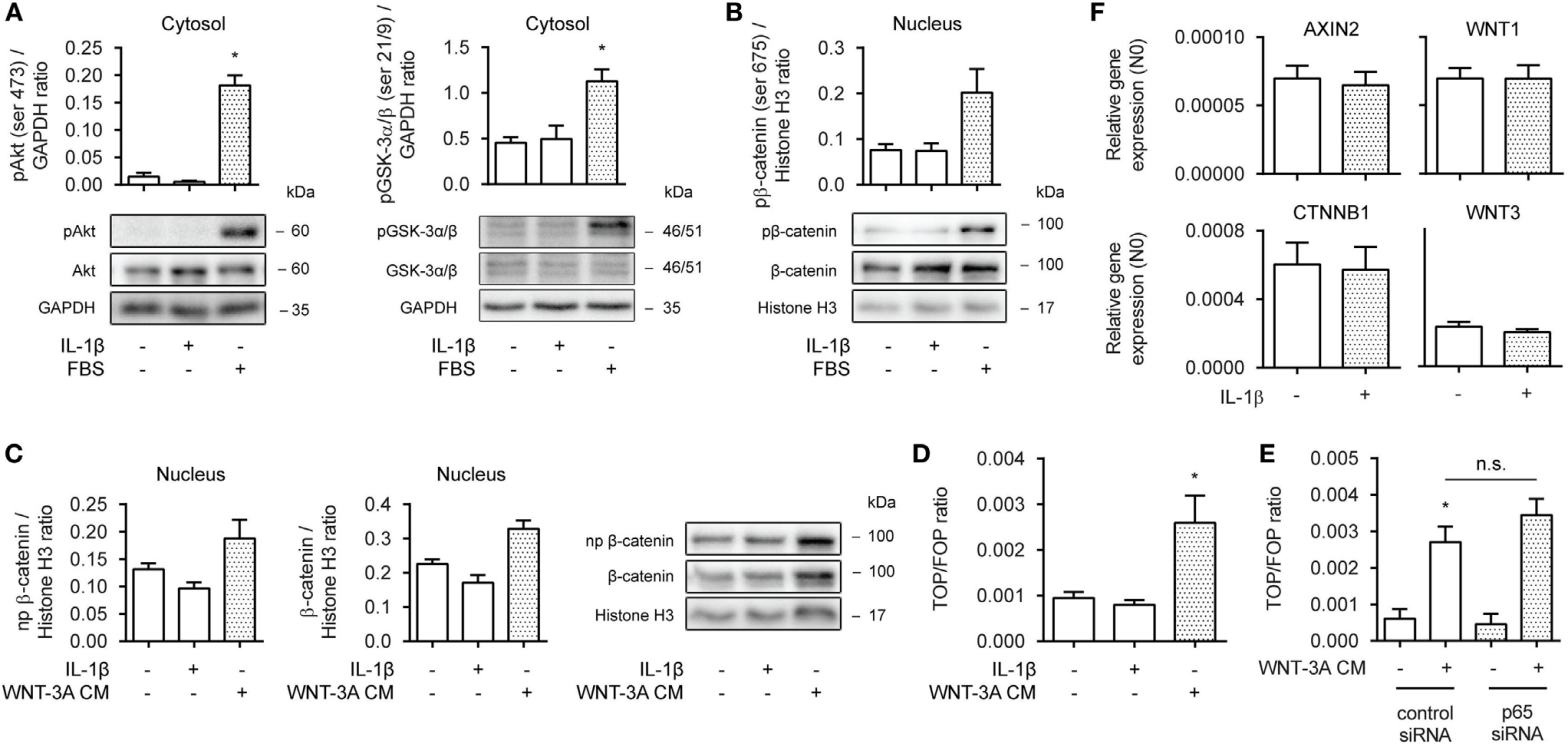
Interleukin-1β (IL-1β) does not promote WNT/β-catenin signaling. **(A)** Cytosolic extracts were used to immunoblot phospho (ser 473) Akt (left panel) and phospho (ser 21/9) glycogen synthase kinase (GSK)-3α/β (right panel) and normalized against GAPDH. Cells were treated with IL-1β (1 ng/mL) or fetal bovine serum (FBS) (10% v/v) for 30 min. Data represent four independent experiments. **(B)** Nuclear extracts were used to immunoblot phospho (ser 675) β-catenin as in **(A)**. Data represent three independent experiments. **(C)** Nuclear extract immunoblot of non-phospho (active) β-catenin and total β-catenin, normalized to Histone H3. Cells were treated with IL-1β (1 ng/mL) or WNT-3A conditioned medium (100%) for 16 h. Data represent three independent experiments. **(D)** TOPFlash reporter assay of human airway smooth muscle cells exposed to IL-1β (1 ng/mL) or WNT-3A conditioned medium (100%) for 24 h. Data represent four independent experiments. **(E)** TOPFlash reporter assay of human airway smooth muscle exposed to WNT-3A conditioned medium (100%) while transiently transfected with 30 pmol control or p65 small-interfering RNA (siRNA). Data represent seven independent experiments. **(F)** mRNA of airway smooth muscle cells exposed to IL-1β (1 ng/mL) for 24 h was isolated and subjected to RT-qPCR for canonical WNT-target genes. Data represent three independent experiments. * vs. control.

To learn more on how β-catenin facilitates IL-1β-induced expression of IL-6, we first asked whether the observed effects were due to enhanced β-catenin activation and target gene expression. In human ASM, β-catenin can be stabilized through both WNT-dependent and independent mechanisms (33), either *via* WNT proteins or growth factors such as PDGF or TGF-β. Cytosolic extracts of 30 min IL-1β-treated cells did not show enhanced ser473 phosphorylation levels, indicative of active Akt, or of phospho GSK-3α/β (ser21/9), indicative of its inhibition (Figure 2A, left and right panel). Similarly, PKA has been shown to phosphorylate β-catenin at ser675, inducing its accumulation in the nucleus and transcriptional activity (15). However, we found no evidence that IL-1β increased levels of phospho ser675 β-catenin in nuclear extracts, following a 30 min treatment (Figure 2B). As expected, the positive control FBS did result in enhanced levels of phospho Akt, GSK-3, and β-catenin (Figures 2A,B). Next, we treated human ASM with WNT-3A cm for 16 h to stabilize β-catenin through WNT-dependent effectors. 16 h was chosen, because WNT-driven stabilization of β-catenin relies on inhibition of the destruction complex and passive build-up and nuclear translocation of β-catenin due to *de novo* synthesis. While WNT-3A cm increased the nuclear presence of both non-phospho and total levels of β-catenin, IL-1β failed to do so (Figure 2C). IL-1β also failed to act on β-catenin-mediated TCF-dependent gene transcription, as observed through a TOPFlash reporter assay (Figure 2D). In line with this, inhibition of p65 with siRNA failed to modulate WNT-3A cm-induced TOPFlash activity (Figure 2E). Moreover, PCR analyses revealed no difference in mRNA abundance for the β-catenin target genes AXIN2, WNT-1, CTNNB1 (the gene encoding β-catenin) and WNT-3 following a 24 h treatment with IL-1β (Figure 2F). In conclusion, in human ASM, IL-1β does not promote WNT/β-catenin signaling to regulate IL-6 expression.

### β-Catenin Is Required for p65 Nuclear Translocation

**Figure 3:**
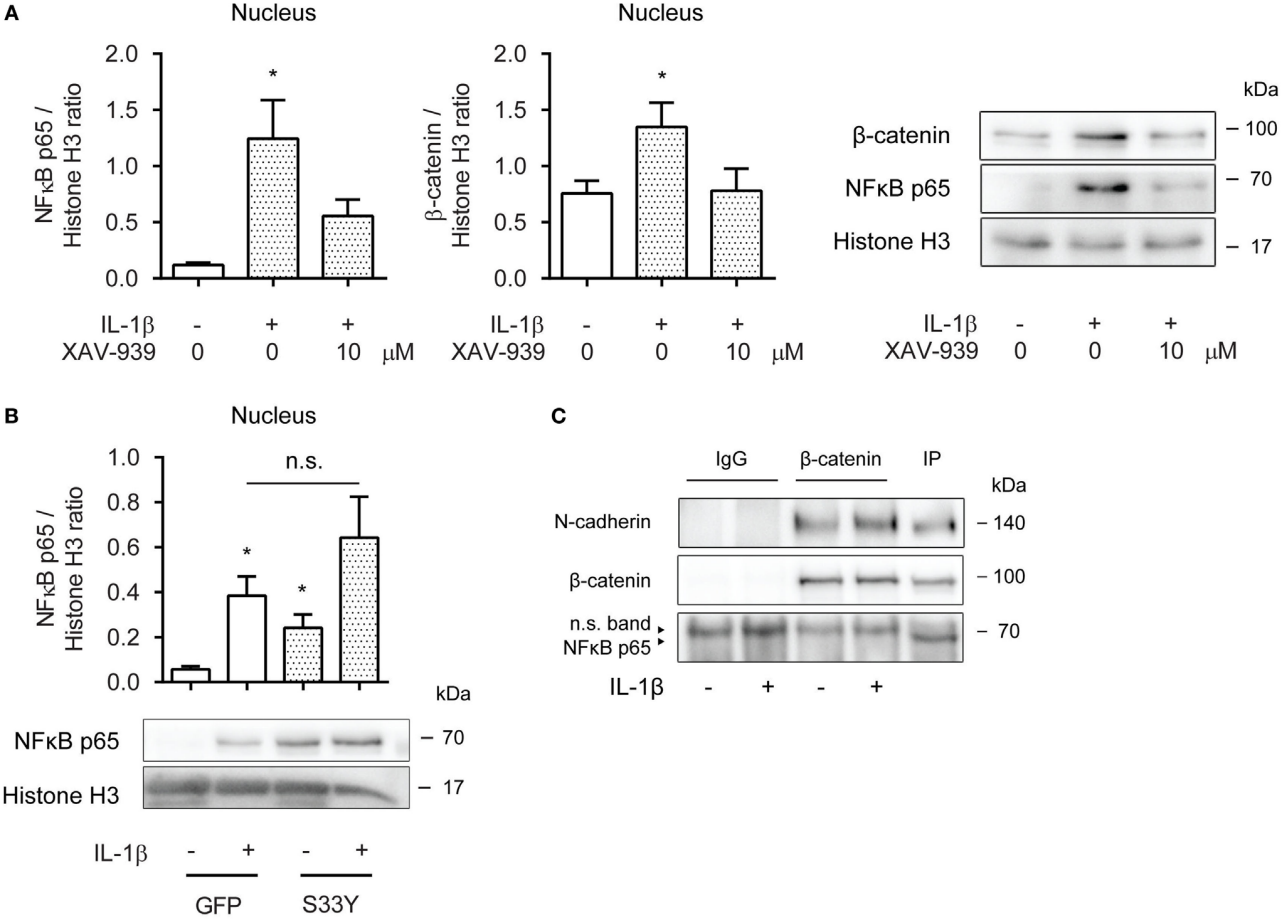
β-Catenin is required for p65 nuclear translocation. **(A)** Nuclear extracts were used to immunoblot nuclear factor-κB (NF-κB) p65 and β-catenin and normalized against Histone H3. Cells were pretreated with XAV-939 (10 µM) or DMSO for 16 h, after which they were stimulated with IL-1β (1 ng/mL) for 30 min. Data represent six independent experiments. **(B)** Nuclear extracts were used to immunoblot NF-κB p65 and normalized against Histone H3. Cells were transiently transfected with a green fluorescent protein (GFP) or S33Y-construct for 24 h, and then treated with IL-1β for 30 min. Data represent nine independent experiments. **(C)** Human airway smooth muscle cells were exposed to IL-1β (1 ng/mL) for 30 min. Stimulated lysates were subjected to immunoprecipitation using a β-catenin specific antibody and mouse IgG as control and were subsequently subjected to SDS-PAGE followed by Western blot using the indicated antibodies. * vs. control.

Next, we asked whether cytosolic β-catenin was required for nuclear translocation of p65. Nuclear extracts obtained from ASM cells pretreated with XAV-939 for 16 h and then exposed to IL-1β for 30 min revealed diminished levels of p65 compared to cells not treated with XAV-939 (Figure 3A). Interestingly, while we initially failed to find increased levels of β-catenin in the nucleus after 16 h of IL-1β treatment (Figure 2C), we did observe increased nuclear presence of β-catenin after 30 min of IL-1β treatment (Figure 3A), suggesting IL-1β promotes rapid shuttling of β-catenin that has already been synthesized in a WNT-independent manner. As XAV-939 promotes degradation of global levels of β-catenin, the observed nuclear translocation following exposure to IL-1β could almost be completely prevented by XAV-939 pretreatment (Figure 3A). Similarly, transfection with the S33Y-mutant resulted in a trend toward enhanced levels of p65 in nuclear extracts (Figure 3B). Of note, S33Y-transfected cells showed increased nuclear presence of p65 even without prior IL-1β exposure (Figure 3B). As both β-catenin and p65 translocate to the nucleus following IL-1β treatment, we hypothesized that β-catenin physically interacts with the p65 subunit of NF-κB. This has also been reported in literature (19–21). Next, we immunoprecipitated β-catenin and performed Western blotting. Interestingly, NF-κB p65 failed to coimmunoprecipitate with β-catenin (Figure 3C) after 30 min IL-1β treatment. The positive control N-cadherin, a transmembrane protein that stably interacts with β-catenin in the plasma membrane did coimmunoprecipitate as expected (Figure 3C). Together, the data show that β-catenin is required for p65 nuclear translocation. While β-catenin colocalizes with p65 in the nucleus in IL-1β-treated cells, they do not physically interact directly.

### CBP and p300 Convey IL-1β-Responsiveness *via* β-Catenin

**Figure 4:**
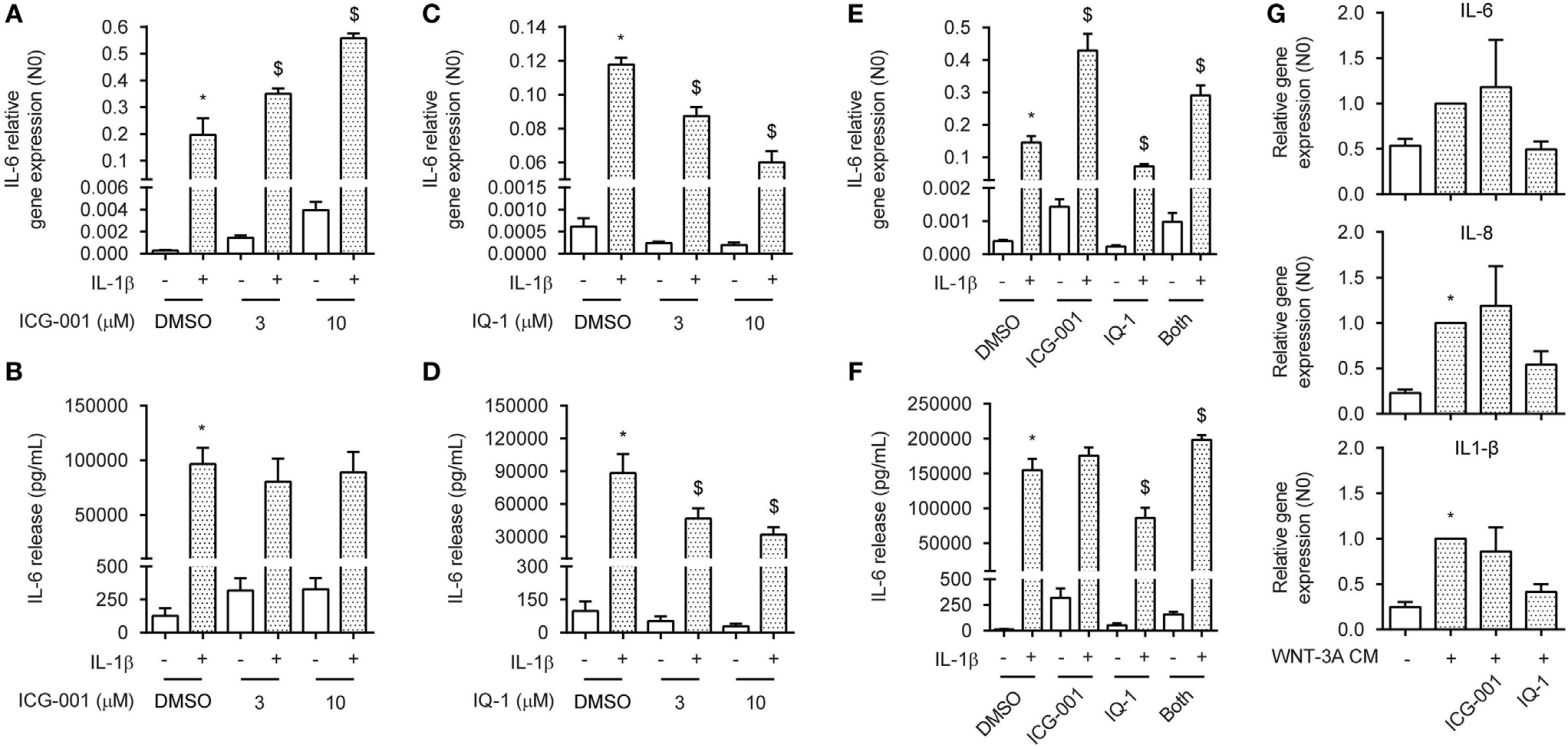
CREB-binding protein (CBP) and p300 convey interleukin-1β (IL-1β)-responsiveness *via* β-catenin. **(A)** IL-6 mRNA and **(B)** secreted protein from airway smooth muscle cells exposed to IL-1β (1 ng/mL) for 16 h **(A)** or 24 h **(B)** with or without ICG-001 (3 and 10 µM). RNA was isolated and subjected to RT-qPCR **(A)** whereas cell supernatants were subjected to enzyme-linked immunosorbent assay (ELISA) **(B)**. Data represent four **(A)** and six **(B)** independent experiments. **(C)** IL-6 mRNA and **(D)** secreted protein as in **(A,B)**, but treated with IQ-1 (3 and 10 µM). Data represent four **(C)** and six **(D)** independent experiments. **(E)** IL-6 mRNA and **(F)** secreted protein as in **(A,B)**, but treated with ICG-001 (10 µM), IQ-1 (10 µM) or the combination. Data represent four **(E)** and six **(F)** independent experiments. **(G)** IL-6, IL-8, and IL-1β mRNA of airway smooth muscle exposed to WNT-3A conditioned medium (100%) for 18 h with or without DMSO, ICG-001 (10 µM), or IQ-1 (10 µM). Data represent seven independent experiments. * vs. control and $ vs. IL-1β.

In the nucleus, both CBP and p300 have been shown to interact with p65 through binding and acetylation, affecting p65 nuclear shuttling, DNA binding affinity or transcriptional activity (31, 32, 41). The ASM actively utilizes CBP and p300 through binding with β-catenin, regulating both ASM proliferation and deposition of extracellular matrix components (33). To determine whether β-catenin binding to either CBP or p300 was a necessary event in the signal regulation of IL-1β-induced expression of IL-6, we treated ASM cells with the small molecule compounds ICG-001 or IQ-1. ICG-001 selectively inhibits the β-catenin/CBP interaction, without affecting the β-catenin/p300 interaction (42). Contrary, IQ-1 prevents the β-catenin/p300 interaction by reducing p300 phosphorylation through inhibition of the protein phosphatase 2A (35). Interestingly, we found that treatment with ICG-001 together with IL-1β dose dependently increased mRNA expression of IL-6 in human ASM cells, but had no effect on protein level (Figures 4A,B). Treatment with ICG-001 alone without IL-1β exposure also increased IL-6 production, albeit to a much lesser extent compared to when IL-1β was added (Figures 4A,B). We found the opposite effect for IQ-1, which dose dependently reduced IL-6 production that was induced by IL-1β, for both protein and mRNA (Figures 4C,D). Again, basal IL-6 levels were also reduced following IQ-1 treatment. Interestingly, for both mRNA and protein, adding both compounds simultaneously largely normalized the stimulatory/inhibitory response induced either by ICG-001 or by IQ-1 (Figures 4E,F). We hypothesized that stabilization of β-catenin through WNT-effectors in combination with ICG-001 or IQ-1 would have to mimic these results. Indeed, similar to what we observed before (Figure 1C), WNT-3A cm increased the expression of the IL-1β target genes IL-6, IL-8, and IL-1β (Figure 4G). While the response of ICG-001 was highly variable, addition of IQ-1 consistently inhibited WNT-3A cm-induced expression of IL-6 (Figure 4G). These results are likely due to a small pool of free nuclear p65 that is present under basal conditions, as the WNT-3A cm effects on IL-6 expression are several folds lower compared to IL-1β. Taken together, CBP and p300 regulate IL-1β-induced expression of IL-6 in human ASM cells through β-catenin.

### β-Catenin Modulates p65 Acetylation on Lysine 310 through Binding with p300

**Figure 5:**
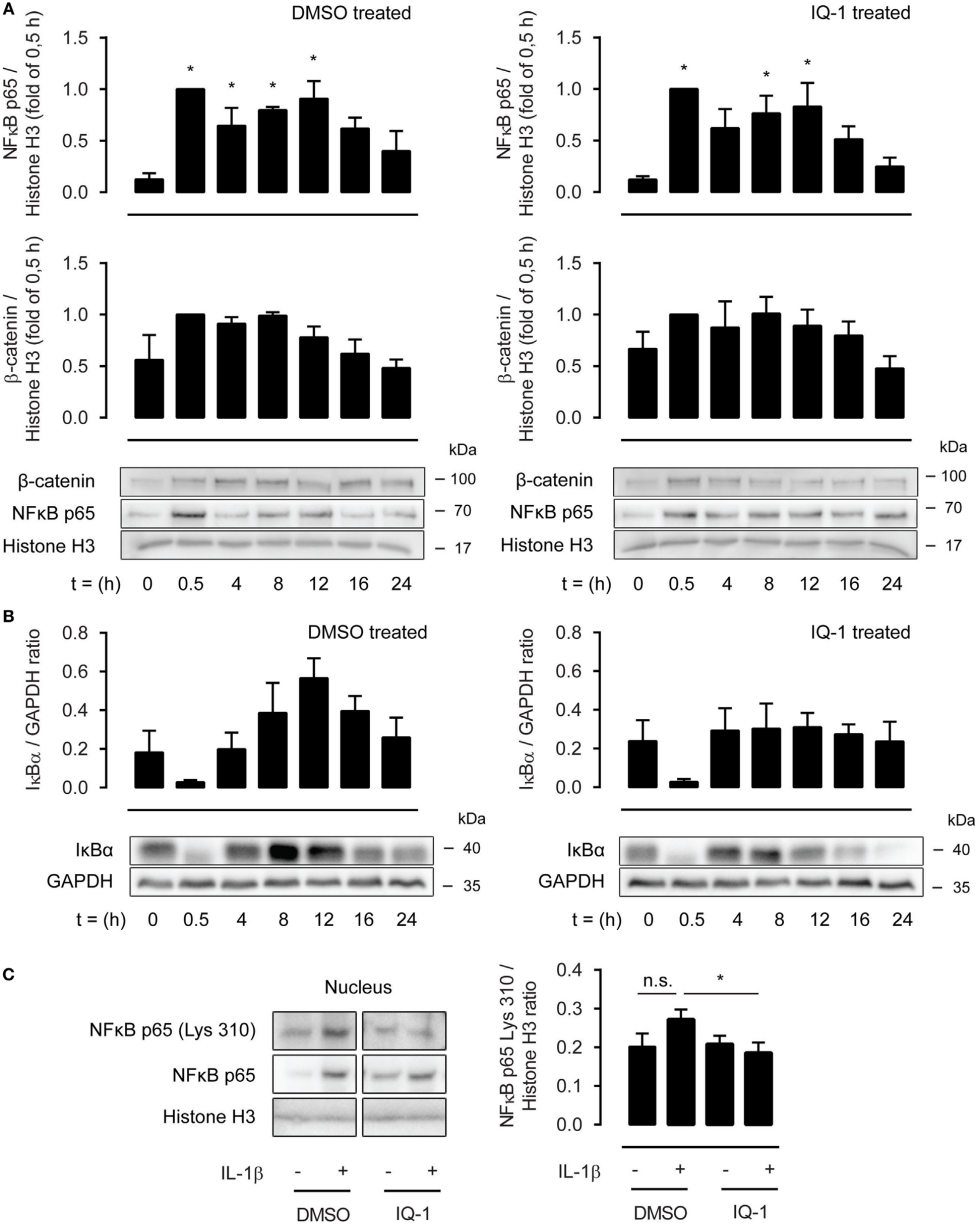
β-Catenin modulates p65 acetylation through p300. **(A)** Nuclear extracts were used to immunoblot nuclear factor-κB (NF-κB) p65 and β-catenin, normalized against Histone H3, at different time points following IL-1β (1 ng/mL) stimulation, and pretreated for 30 min with DMSO (left panel) or IQ-1 (right panel). Data represent five independent experiments. **(B)** Cytosolic extracts were used to immunoblot IκB-α and normalized against GAPDH, as in **(A)**. Data represent five independent experiments. **(C)** Nuclear extract immunoblot of acetylated p65 (lys 310), normalized against Histone H3. Airway smooth muscle cells were treated with IL-1β (1 ng/mL) for 30 min with IQ-1 or DMSO as a vehicle control. Data represent six independent experiments. * vs. control.

As the effects of IQ-1 were more robust compared to ICG-001, we next investigated the underlying mechanisms of action focusing on p300. p300 and CBP are histone acetyl transferases and can acetylate p65 at lysine 221, impairing p65-IκB-α assembly while enhancing DNA binding. This results in increased nuclear export mediated by IκB-α. We treated ASM cells with IL-1β up to 24 h in the presence or absence of IQ-1 or DMSO as a vehicle control. However, nuclear extracts revealed no differences in p65 abundance between both groups (Figure 5A). In line with this, the temporal profile for nuclear β-catenin was also identical for IQ-1 and DMSO-treated cells (Figure 5A). Interestingly, p65 and β-catenin nuclear translocation showed a biphasic response following IL-1β treatment with a secondary peak around 8–12 h. We also studied the cytosolic presence of IκB-α. As expected, IκB-α degradation occurred rapidly following IL-1β treatment and levels were restored completely again after 4 h. However, there was no difference in expression between IQ-1 and DMSO treatment (Figure 5B). IκB-α also displayed a secondary peak, which was significantly enhanced in the DMSO-treated group, but mostly absent in the IQ-1 group (Figure 5B). Next, we studied p65 acetylation of lysine 310, which is required for full transcriptional activity (41). Nuclear extracts from ASM cells stimulated with IL-1β for 30 min revealed that IL-1β induced a trend toward increased p65 acetylation of lysine 310, while IQ-1 pretreatment completely prevented this (Figure 5C). Taken together, the results suggest β-catenin mediates NF-κB p65 signaling by facilitating acetylation of p65 at lysine 310 by p300, which likely inhibits transcriptional activity of p65. Although we cannot exclude the possibility that IQ-1 also affects acetylation of lysine 221, failure to detect differences in nuclear shuttling suggests that this may not be the case.

## Discussion

**Figure 6:**
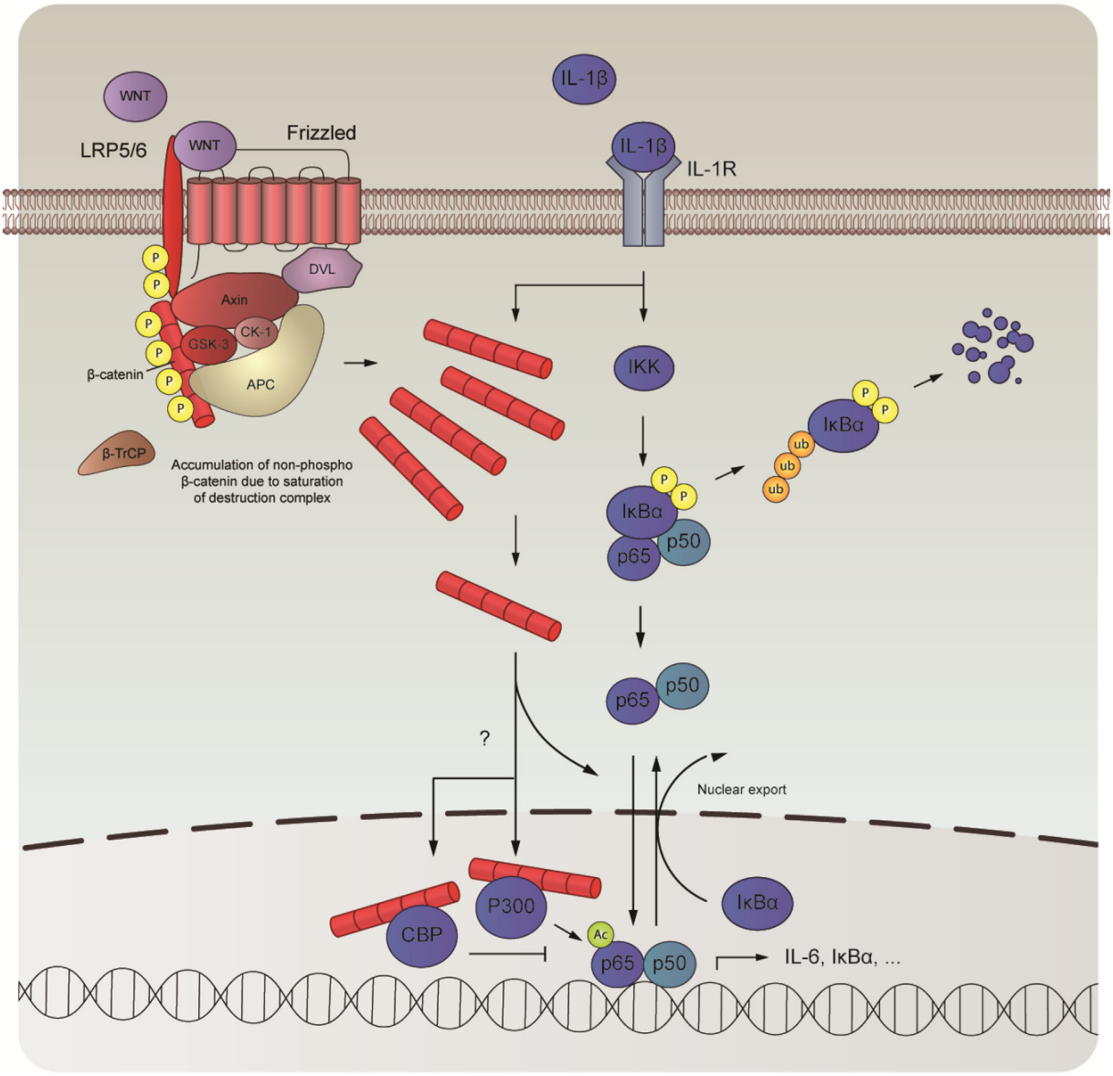
Schematic overview of the interaction between nuclear factor-κB (NF-κB) and β-catenin. In human airway smooth muscle, interleukin-1β (IL-1β) drives the expression of proinflammatory genes, such as IL-6, by interacting with the WNT-effector β-catenin. Although IL-1β does not directly influence expression levels of β-catenin, it directs its nuclear import together with the NF-κB dimer to facilitate DNA-binding of p65. Inside the nucleus, β-catenin teams up with either CREB-binding protein (CBP) or P300, two mutually exclusive events that are necessary for transcriptional output by p65. While direct competition between CBP and P300 likely underlies binding with β-catenin, alternative mechanisms may also be at play. When bound, p65 is acetylated, which likely governs its subsequent transcriptional program.

In this study, we examined the role of β-catenin in NF-κB-mediated inflammation in human ASM cells. We observed that β-catenin signaling is required for the expression of IL-6 induced by IL-1β and that elevated levels of intracellular β-catenin increase IL-1β-responsiveness. We further show that IL-1β fails to promote WNT/β-catenin signaling and that the effects on IL-6 are not due to TCF/LEF engagement. Instead, we found evidence that β-catenin acts as a scaffold, providing a critical bridging step in the NF-κB signal transduction pathway mediated by IL-1β. Although we found no evidence that β-catenin engaged in direct physical contact with p65 in human ASM cells, degradation of β-catenin following pretreatment with XAV-939 attenuated p65 nuclear translocation after treatment with IL-1β. In addition, recombinant IL-1β also induced translocation of β-catenin into the nucleus concomitant with p65. In the nucleus, β-catenin pairs up with the cofactors CBP or p300. We observed that CBP and p300 governed p65 signaling output. Inhibition of the β-catenin/CBP interaction amplified IL-6 mRNA expression mediated by IL-1β, whereas inhibition of the β-catenin/p300 attenuated it. We suspect that these changes are due to a change in acetylation of p65 by p300 as treatment with IQ-1 normalized IL-1β’s effects on p65 acetylation at lysine 310 (Figure 6).

Nuclear presence of p65 and β-catenin following treatment with IL-1β up to 24 h revealed a biphasic response, with an initial peak around 0.5 h and a subsequent peak around the 8 to 12 h time point. These results suggest that NF-κB drives a cascade of proinflammatory events that initiates an autocrine loop of NF-κB activation. In this regard, it is interesting to note that there was no difference in p65/β-catenin nuclear presence between IQ-1 and DMSO-treated cells. Because IQ-1 prevented p65 acetylation at lysine 310, thereby reducing transcriptional activation of NF-κB, these results suggest that there may be a subset of IL-1β target genes involved in proinflammatory events that is not under the control of p300. This view is supported by literature, showing that CBP/p300 is sensitive to some, but not all transcriptional events driven by IL-1β (32). Of note, IκB-α synthesis also showed a seemingly compensatory effect, significantly increasing cytoplasmic levels following the initial wave of NF-κB activation. This compensatory effect was mostly lost following treatment with IQ-1. As NF-κB promotes IκB-α expression (43), IQ-1 most likely inhibits p65-mediated expression of IκB-α.

Our results further demonstrate that β-catenin mediates its effects primarily in the nucleus by interacting with either CBP or p300. CBP and p300 are paralogous multidomain proteins that share a large degree of structural similarity. Yet, they are often ascribed opposing functions (35), which is supported by our findings. The molecular events regulating β-catenin binding with either CBP or p300 remain elusive and it has been proposed that the interaction results from competition (35). This would indicate that relative concentration of the cofactors dictates β-catenin binding and therefore transcriptional output. There is some support for this view. Mice nullizygous for p300 die during embryonic development, whereas mice that lacked only one functional allele of the p300 gene, and therefore had approximately half of the normal amount of p300 protein, also die early during embryogenesis (44). At the same time, overexpression of CBP in *Drosophila* (dCBP) embryos results in early death (45). This suggests that the total amount of functional CBP/p300 protein is critical for normal development. In a recent article, it was shown that in MRC-5 lung fibroblasts, inhibition of β-catenin attenuated expression of IL-1β target genes. It was further shown that siRNA targeted against CBP or p300 would inhibit or amplify this response, respectively (32). These results are directly opposite to what we report here and it would be interesting to assess the differences in CBP/p300 concentration between lung fibroblasts and ASM cells. We propose that following a proinflammatory stimulus, β-catenin teams up with the cofactor that is the most abundantly expressed. Inhibition of this interaction will automatically favor binding with the other cofactor, regardless of its concentration, and may result in a response that is opposite compared to the original event. This balance allows the cell to dictate its behavior by shifting the balance between CBP and p300.

Previous reports showed that β-catenin activation inhibits NF-κB signaling through direct binding of β-catenin with the NF-κB subunit p65 (19–21). While we found no evidence for direct binding of β-catenin with p65, failure of p65 to translocate to the nucleus while global levels of β-catenin are low, suggests that complex formation may still occur. It is possible that in human ASM cells, β-catenin interacts with the p50 subunit (19, 20, 22–24). NF-κB proteins form numerous homo- and heterodimers, of which the NF-κB p65/p50 heterodimer is most abundantly expressed. Heterodimers shuttle between cytosol and nucleus as a complex and we cannot exclude the possibility that β-catenin interaction with p50 is necessary for this event.

The results obtained in this study may be relevant for potential therapy development within the context of chronic airway disease. The usefulness of targeting NF-κB in these conditions has already been demonstrated by the efficacy of glucocorticosteroids, which can largely be contributed to inhibition of NF-κB (46). However, a proportion of patients display glucocorticoid resistance. Steroid resistance in asthmatic patients appears to be more common within families, suggesting that genetic factors can determine corticosteroid responsiveness (47), and the mechanisms involved appear heterogeneous (48). In COPD patients, increased oxidative stress has been suggested to play a major role in directing steroid resistance (48). Regardless of its origins, however, many of the features are similar and can essentially be reduced to two main processes, referred to as transrepression and transactivation, both involving NF-κB. Transrepression is the consequence of decreased histone deacetylase (HDAC) expression and activity. This can be caused by a variety of triggers, e.g., increased levels of oxidative or nitrative stress following exposure to cigarette smoke, severe inflammation, or viral infection (48, 49). Defective HDAC expression loosens chromatin structure, allowing for the transcription of otherwise silent inflammatory genes (50, 51). In addition, the ligand-bound glucocorticoid receptor (GR) remains acetylated for longer, preventing its association with NF-κB in the nucleus (52). Transactivation involves direct DNA binding by ligand-bound GR dimers. For example, glucocorticoids are well known for their stimulatory effects on IκBα, including in airway structural cells like ASM (53–55). Many of the negative side effects of long-term glucocorticoid treatment are thought to occur as a result of transactivation, thus there has been considerable interest in developing transrepression-selective glucocorticoids. In steroid-resistant patients receiving these kinds of drugs, targeting the NF-κB pathway may be useful both as replacement and add-on therapy. Where transactivation is concerned, targeting NF-κB may be best considered as an individual therapy. Targeting the β-catenin/CBP/p300 interaction may be interesting, as it only targets a very specific subset of NF-κB target genes. In addition, it avoids potential side effects caused by direct targeting of NF-κB in off-target tissues. For example, NF-κB activation in human neutrophils regulates apoptosis (56). Before we can safely consider the β-catenin/CBP/p300 interaction as a viable treatment strategy, it is essential that we gain more insight into what determines β-catenin binding with either CBP or p300 and how this affects transcriptional output of interacting proteins like NF-κB. In this study, CBP and p300 mediate opposite effects driven by NF-κB p65. In light of the broad actions of β-catenin, some airway diseases may be more suited for β-catenin directed therapy than others. Asthma is generally associated with elevated levels of β-catenin or canonical WNT signaling effectors, thus a targeted approach may act beneficial beyond NF-κB. For COPD, β-catenin or canonical WNT mediators are deficiently expressed in various lung compartments. Blocking β-catenin output may therefore yield unwanted effects (57). These points will have to be addressed.

In conclusion, we show that in human ASM β-catenin signaling is required for IL-1β-induced expression of IL-6. Here, β-catenin acts as a scaffold to compartmentalize NF-κB signaling by interacting with CBP and p300. We cannot state with certainty what the relative contribution of each cofactor is, but our results point toward an active role for p300 in mediating p65 acetylation and transcriptional output. The role of CBP is less clear. It may serve as an inhibitor or is not actively involved in the regulation of IL-6. Detailed insights into how β-catenin bridges p300 with NF-κB signaling remains to be elucidated and is an important next step toward a possible therapeutic application.

## Author Contributions

TK conceived the study and designed the experiments, carried out the experiments, analyzed the data, and drafted the manuscript. MM and RE carried out the experiments. RG conceived the study and designed the experiments. AH provided the human ASM cells essential for this study. All authors critically revised the manuscript for important intellectual content and approved its final version.

## Conflict of Interest Statement

The authors declare that the research was conducted in the absence of any commercial or financial relationships that could be construed as a potential conflict of interest.

## Abbreviations

ASM, airway smooth muscle; B2M, beta-2-microglobulin; COPD, chronic obstructive pulmonary disease; CBP, CREB-binding protein; p300, E1A-associated protein p300; FBS, fetal bovine serum; GSK-3, glycogen synthase kinase-3; hTERT, human telomerase reverse transcriptase; IL-1, interleukin-1; IL-1β, interleukin-1β; NF-κB, nuclear factor-κB; PDGF, platelet-derived growth factor; PKA, protein kinase A; RPL13A, ribosomal protein L13A; TCF/LEF1, T-cell factor/lymphoid enhancer-binding factor 1; TLR, toll-like receptor; TGF-β, transforming growth factor beta; TNF-α, tumor necrosis factor-alpha.
